# Angioscopic Evaluation of Thrombi in the Culprit Coronary Lesions in Patients With Acute Myocardial Infarction

**DOI:** 10.1155/DTE.7.1

**Published:** 2000

**Authors:** H. Morio, Y. Fujimori, K. Terasawa, O. Hasegawa, A. Matsuo, M. Osegawa

**Affiliations:** Department of Cardiology Narita Red Cross Hospital 90-1 Iida-cho Narita city, Chiba 286-8523 Japan

## Abstract

The purpose of this study was to evaluate intracoronary thrombi in the culprit lesions in patients with acute myocardial infarction (AMI) by angioscopy, and to compare them with clinical and angiographic features. We angioscopically observed the culprit coronary lesions in 66 patients with AMI (55 males and 11 females, 63.9±15.4 years old) just before interventional therapy. Thrombi were observed in 42 of 66 lesions (64%), namely, red thrombi in 16, mixed thrombi in 15, white thrombi in 11. In patients with complete obstruction (TIMI grade 0 and I), red thrombi were more frequently observed than mixed or white thrombi. On the other hand, in patients with incomplete obstruction (TIMI grade II and III), white thrombi were more frequently observed than the others. Angiographically, haziness and filling defect were significantly more frequently observed in patients with red thrombi than the others (p<0.05). The distance from proximal side branch to thrombi tended to be longer in patients with red thrombi than the others. The time from onset of AMI tended to be longer in patients with white thrombi than the others. These results suggest that blood flow may be an important determinant of thrombi characterization.


*Diagnostic and Therapeutic Endoscopy*, Vol. 7, pp. 1–5
Reprints available directly from the publisher
Photocopying permitted by license only

© 2000 OPA (Overseas Publishers Association) N.V.
Published by license under
the Harwood Academic Publishers imprint,
part of The Gordon and Breach Publishing Group.
Printed in Malaysia.

### Angioscopic Evaluation of Thrombi in the Culprit Coronary Lesions in Patients with Acute Myocardial Infarction

H. MORIO*, Y. FUJIMORI, K. TERASAWA, O. HASEGAWA, A. MATSUO and M. OSEGAWA

*Department of Cardiology, Narita Red Cross Hospital, 90-1, Iida-cho, Narita City, Chiba, 286-8523, Japan*

*(Received 27 April 2000; Revised 29 May 2000; In final form 29 May 2000)*

**The purpose of this study was to evaluate intracoronary thrombi in the culprit lesions in patients with acute myocardial infarction (AMI) by angioscopy, and to compare them with clinical and angiographic features. We angioscopically observed the culprit coronary lesions in 66 patients with AMI (55 males and 11 females, 63.9 ± 15.4 years old) just before interventional therapy. Thrombi were observed in 42 of 66 lesions (64%), namely, red thrombi in 16, mixed thrombi in 15, white thrombi in 11. In patients with complete obstruction (TIMI grade 0 and I), red thrombi were more frequently observed than mixed or white thrombi. On the other hand, in patients with incomplete obstruction (TIMI grade II and III), white thrombi were more frequently observed than the others. Angiographically, haziness and filling defect were significantly more frequently observed in patients with red thrombi than the others ($p < 0.05$). The distance from proximal side branch to thrombi tended to be longer in patients with red thrombi than the others. The time from onset of AMI tended to be longer in patients with white thrombi than the others. These results suggest that blood flow may be an important determinant of thrombi characterization.**

*Keywords*: Acute myocardial infarction, Angioscopy, Coronary thrombi, Culprit coronary lesion

### INTRODUCTION

Recent pathological studies revealed that plaque disruption and subsequent local thrombosis were the main clue events in acute myocardial infarction (AMI), resulting in coronary flow reduction and myocardial damages [1]. Morphological changes in the coronary arteries in patients with AMI had been clinically evaluated by coronary angiography [2]. Although a powerful diagnostic tool, angiography can display only the silhouette of the luminal changes and is not helpful enough to investigate the pathogenesis of AMI. Therefore, development of a new diagnostic method which gives us more

* Corresponding author. Tel.: +81-476-22-2311. Fax: +81-476-22-8024. E-mail: morio-chan@nifty.ne.jp detailed and precise pathological information is required. Angioscopy, which enables direct visualization of endovascular surface, is a more accurate means of identifying pathological features of the plaques in patients with ischemic heart disease [3,4,5,6]. Several angioscopic observations in patients with AMI had revealed high frequency of plaque disruption and subsequent thrombosis in the coronary lesions [7,8,9,10]. Coronary thrombi observed by angioscopy are classified three types, red, white and mixed thrombi [8,10]. It was reported that patients with unstable angina were frequently observed to have white thrombi, but none were seen in the patients with AMI, whereas red thrombi were frequently observed in patients with AMI [8]. Our findings about colors of thrombi are different from this report. Moreover, the difference between the mechanism of the formation of white thrombi and that of red thrombi has not been known. To clarify these differences, we angioscopically observed the culprit coronary lesions and compared them with clinical and angiographic features.

### PATIENTS AND METHODS

### Patients

From December 1996 to August 1998, we angioscopically observed the culprit coronary lesions in 66 patients with AMI (55 males and 11 females, $63.9 \pm 15.4$ years old) just before interventional therapy. None of them had shock, congestive heart failure, and ventricular arrhythmia (Lown > III).

### Angioscopy System

Angioscopes used were Vecmova (Clinical Supply Co., Ltd. Gifu, Japan), which were 4.5 F in external diameter. The angioscope had an inflatable balloon located close to the distal most tip and had two lumens, one for 1.5 F fiberscope and saline flash and the other for a guide wire. Since steerable, the fiberscope can be advanced or pulled back for 7 cm during balloon inflation and saline flash.

### Procedures

Details of angioscopy were described elsewhere [3,4]. In brief, after routine coronary angiography, patients received 8000–10000 IU of heparin intravenously, and coronary injection of 0.1 mg of nitroglycerine. A 8 F guiding catheter for PTCA was introduced through the right femoral artery into the coronary artery. Then, an angioscope was introduced over a 0.014 inch guide wire into the coronary artery to locate the angioscope tip just proximal to the culprit plaque. Then, the balloon was inflated with carbon dioxide to interrupt blood flow and heparinized (10 IU/ml) body temperature saline was injected at 0.5 ml/second for observation. During observation, depression or elevation of ST segment, inversion of T wave and elongation of QT segment on electrocardiogram always occurred. When ST elevation appeared, the balloon was deflated for blood flow restoration. Therefore, each observation time was usually around 10 seconds. We obtained informed consent from all patients before examination.

### Statistics

Data are reported as mean $\pm$ SD for continuous data and percentages for discrete data. Comparisons were made with student's $t$ test or $\chi^2$ test, as appropriate. A $p$ value $< 0.05$ was considered statistically significant.

### RESULTS

Thrombi were observed in 42 of 66 lesions (64%), namely, red thrombi in 16, mixed thrombi in 15, white thrombi in 11 (Fig. 1). Distribution of the culprit coronary lesions was left anterior descending artery in 31, left circumflex artery in 10, and right coronary artery in 25 patients.

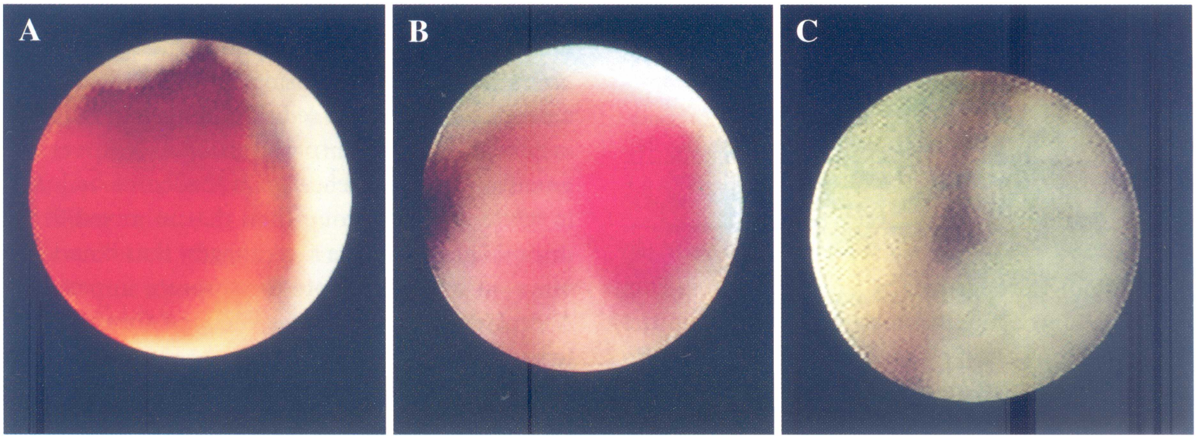

FIGURE 1 Angioscopic findings of thrombi in the culprit coronary lesions in patients with acute myocardial infarction. (A) Red thrombus, (B) Mixed thrombus, (C) White thrombus.

Relation between the lesions distribution and thrombi appearance is listed in Table I. No relation was observed between the lesions distribution and thrombi appearance.

Relation between thrombi appearance and angiographic features is listed in Table II. In patients with complete obstruction (TIMI grade 0 and I), red thrombi were more frequently observed than mixed or white thrombi. On the other hand, in patients with incomplete obstruction (TIMI grade II and III), white thrombi were more frequently observed than red or mixed thrombi. Angiographically, haziness and filling defect were significantly more frequently observed in patients with red thrombi than in patients with mixed and white thrombi ($p < 0.05$, vs. mixed and white). The distance from proximal side branch to thrombi tended to be longer in patients with red thrombi than that in patients with mixed or white thrombi.

TABLE I Relation between the lesions distribution and the thrombi as detected by angioscopy

| Lesions distribution | Thrombi as detected by Angioscopy | | | |
|---|---|---|---|---|
| | red ($n=16$) | mixed ($n=15$) | white ($n=11$) | absence ($n=24$) |
| LAD ($n=31$) | 7 | 6 | 5 | 13 |
| LCX ($n=10$) | 1 | 1 | 3 | 5 |
| RCA ($n=25$) | 8 | 8 | 3 | 6 |

TABLE II Relation between the angiographic appearance and the thrombi as detected by angioscopy

| Angiographic appearance | Thrombi as detected by Angioscopy | | | |
|---|---|---|---|---|
| | red ($n=16$) | mixed ($n=15$) | white ($n=11$) | absence ($n=24$) |
| TIMI grade | | | | |
| O + I (complete obstruction) | 14 | 11 | 5 | 17 |
| II + III (incomplete obstruction) | 2 | 4 | 6 | 7 |
| Haziness, Filling defect | 12* | 5 | 3 | 0 |
| Distance from the proximal side branch (mm) | 8.1 ± 8.0 | 5.6 ± 3.8 | 5.1 ± 3.8 | 4.5 ± 4.8 |

*$p < 0.05$.

Relation between appearance of thrombi and time from onset of chest pain to angioscopy is listed in Table III. The time tended to be longer in white thrombi group than that in mixed or red thrombi group.

### DISCUSSION

In this study, thrombi were frequently (42 of 66 lesions, 64%) observed in the culprit coronary lesions in patients with AMI. Of 42 thrombi, red thrombi in 16, mixed thrombi in 15 and white thrombi in 11 were observed. It was reported that

TABLE III Relation between the time from onset to angiography and the thrombi as detected by angioscopy

| | Thrombi as detected by Angioscopy | | | |
|---|---|---|---|---|
| | red ($n=16$) | mixed ($n=15$) | white ($n=11$) | absence ($n=24$) |
| Time from onset to angiography (hr) | $3.8\pm2.8$ | $4.9\pm4.3$ | $5.2\pm5.1$ | $6.7\pm6.1$ |

red thrombi were frequently observed in patients with AMI, but white thrombi were not seen in the patients with AMI [8]. Our findings about colors of thrombi are different from this report. It was also reported that white thrombi were frequently observed in patients with AMI, but red thrombi were observed in a limited amount in a small fraction of patients with AMI [9]. In their report, they observed the culprit lesions after reperfusion, whereas in our study, observations were performed before interventional therapy. Spontaneous reperfusion (TIMI grade II and III) seen in some patients in our study might account for the difference of the color of thrombi.

Previous angioscopic studies in patients with AMI had not revealed the difference between the mechanism of the formation of white thrombi and that of red thrombi. What is the factor deciding the kind of thrombi? In our study, in patients with complete obstruction, red thrombi were more frequently observed than mixed or white thrombi, whereas in patients with incomplete obstruction, white thrombi were more frequently observed than red or mixed thrombi. The difference in color of thrombi probably reflects difference in the composition of thrombi, perhaps related in part to the different ages of the thrombi or difference of blood flow in the artery [8]. Whereas red thrombi tend to form under conditions of stasis, white thrombi tend to form when blood flow has not been completely interrupted. Pathological studies of coronary thrombi showed that white thrombi were platelet-rich, whereas red thrombi contained an abundance of fibrin mixed with erythrocytes and platelets [11]. In addition, when observed after thrombolysis, white thrombi were found to be older than red thrombi and had a tight fibrin network [12].

In our study, white thrombi were observed even in the patients with complete obstruction. The distance from proximal side branch to thrombi tended to be longer in red thrombi group than that in mixed or white thrombi group. Moreover, the time from onset of chest pain tended to be longer in white thrombi group than that in mixed or red thrombi group. These facts suggest that blood flow plays an important role for thrombi appearance. A probable explanation is that red thrombi were formed in the initial stage of AMI, and erythrocytes in the thrombi might be dispersed by blood flow (sometimes by coronary blood stream to proximal side branches around thrombi), while platelets and fibrins remain, resulting in white in color (Fig. 2). When the proximal side branch is near the thrombus, some part of blood stream to proximal side branch can flow around the thrombus (Fig. 2A). But when the proximal side branch is far from the thrombus, any part of blood stream cannot flow around the thrombus, and cannot disperse erythrocytes in the thrombus (Fig. 2B).

Angiographically, haziness and filling defect were significantly more often observed in patients with red thrombi than in patients with mixed and white thrombi. The findings of haziness and filling defect are angiographic criteria for the presence of intracoronary thrombi [13,14,15], though angiography has been shown to be less sensitive than angioscopy for detection of thrombi [6,7]. Angio-

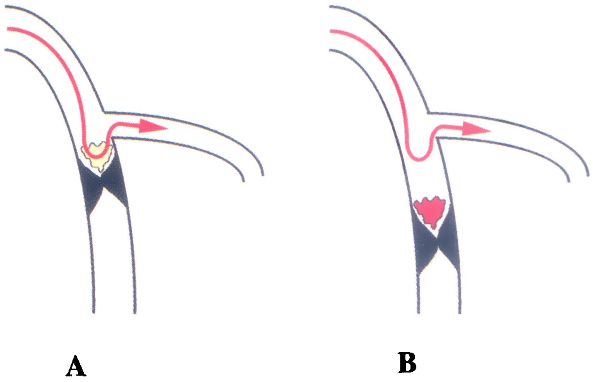

FIGURE 2 Schematic representation of the difference between the mechanism of the formation of white thrombus (A) and that of red thrombus (B).

graphy can probably recognize only the thrombi under conditions of stasis. The findings of haziness and filling defect reflect the silhouette of the stasis of contrast medium. In white thrombi, contrast medium probably can flow through the fibrin network, resulting in lack of the findings of haziness and filling defect.

In conclusion, thrombi formation has an important role in the pathogenesis of AMI. Not only red thrombi but also white and mixed thrombi were observed in the culprit lesions in the patients with AMI. The results in this study suggest that blood flow may be an important determinant of thrombi characterization.

### *References*

[1] Fuster, V., Badimon, L., Badimon, J.J. and Chesebro, J.H. The pathogenesis of coronary artery disease and the acute coronary syndromes. *N. Engl. J. Med.* 1992; **326**: 245–250.

[2] Ambrose, J., Tannenbaum, M., Alexopoulos, D., Hjemdahl-Monsen, C.E., Leavy, J., Weiss, M., Borrico, S., Gorlin, R. and Fuster, V. Angiographic progression of coronary artery disease and the development of myocardial infarction. *J. Am. Coll. Cardiol.* 1988; **12**: 56–62.

[3] Uchida, Y., Tomaru, T., Nakamura, F., Furuse, A., Fujimori, Y. and Hasegawa, K. Percutaneous coronary angioscopy in patients with ischemic heart disease. *Am. Heart. J.* 1987; **114**: 1216–1222.

[4] Uchida, Y., Nakamura, F., Tomaru, T., Morita, T., Oshima, T., Sasaki, M., Morizuki, O. and Hirose, J. Prediction of acute coronary syndrome by percutaneous coronary angioscopy in patients with stable angina. *Am. Heart. J.* 1995; **130**: 195–203.

[5] Mizuno, K., Arai, T., Satomura, K., Shibuya, T., Arakawa, K., Okamoto, Y., Miyamoto, A., Kurita, A., Kikuchi, M. and Nakamura, H. New percutaneous transluminal coronary angioscope. *J. Am. Coll. Cardiol.* 1989; **13**: 363–368.

[6] Ramee, S.R., White, C.J., Collins, T.J., Mesa, J.E. and Murgo, J.P. Percutaneous angioscopy during coronary angioplasty using a steerable microangioscope. *J. Am. Coll. Cardiol.* 1991; **17**: 100–105.

[7] Mizuno, K., Miyamoto, A., Satomura, K., Kurita, A., Arai, T., Sakurada, M., Yanagida, S. and Nakamura, H. Angioscopic coronary macromorphology in patients with acute coronary disorders. *Lancet* 1991; **337**: 809–812.

[8] Mizuno, K., Satomura, K., Miyamoto, A., Arakawa, K., Shibuya, T., Arai, T., Kurita, A., Nakamura, H. and Ambrose, J.A. Angioscopic evaluation of coronary-artery thrombi in acute coronary syndromes. *N. Engl. J. Med.* 1992; **326**: 287–291.

[9] Ueda, Y., Akasaka, M., Hirayama, A., Komamura, K., Hori, M. and Kodama, K. Intracoronary morphology of culprit lesions after reperfusion in acute myocardial infarction: serial angioscopic observations. *J. Am. Coll. Cardiol.* 1996; **27**: 606–610.

[10] Van Belle, E., Lablanche, J.M., Bauters, C., Renaud, N., McFadden, E.P. and Bertrand, M.E. Coronary angioscopic findings in the infarct-related vessel within 1 month of acute myocardial infarction: natural history and effect of thrombolysis. *Circulation* 1998; **97**: 26–33.

[11] Pasternack, R.C., Braunwald, E. and Sobel, B.E. Acute myocardial infarction. In: Braunwald, E., ed. Heart disease: a textbook of cardiovascular medicine. 3rd ed. Vol. 2. Philadelphia: W.B. Saunders, 1988; 1222–1313.

[12] Uchida, Y., Masuo, M., Tomaru, T., Kato, A. and Sugimoto, T. Fiberoptic observation of thrombosis and thrombolysis in isolated human coronary arteries. *Am. Heart. J.* 1986; **112**: 691–696.

[13] Ambrose, J.A., Winters, S.L., Stern, A., Eng, A., Teicholz, L.E., Gorlin, R. and Fuster, V. Angiographic morphology and the pathogenesis of unstable angina. *J. Am. Coll. Cardiol.* 1985; **5**: 609–616.

[14] Levin, D.C. and Fallon, J.T. Significance of the angiographic morphology of localized coronary stenoses: histologic correlation. *Circulation* 1982; **66**: 316–320.

[15] Wilensky, R.L., Bourdillon, P.D.V., Vix, V.A. and Zeller, J.A. Intracoronary artery thrombus formation in unstable angina: a clinical, biochemical and angiographic correlation. *J. Am. Coll. Cardiol.* 1993; **21**: 692–699.

